# Mitochondrial DNA impact on joint damaged process in a conplastic mouse model after being surgically induced with osteoarthritis

**DOI:** 10.1038/s41598-021-88083-0

**Published:** 2021-04-27

**Authors:** Morena Scotece, Ignacio Rego-Pérez, Ana Victoria Lechuga-Vieco, Alberto Centeno Cortés, María Concepción Jiménez-Gómez, Purificación Filgueira-Fernández, Carlos Vaamonde-García, José Antonio Enríquez, Francisco J. Blanco

**Affiliations:** 1 Grupo de Investigación de Reumatología (GIR), Instituto de Investigación Biomédica de A Coruña (INIBIC), Complexo Hospitalario Universitario de A Coruña (CHUAC), Sergas, Universidade da Coruña (UDC), A Coruña, Spain; 2 Centro Nacional de Investigaciones Cardiovasculares Carlos III, Madrid, Spain; 3 CIBERES, C/Melchor Fernández-Almagro 3, 28029 Madrid, Spain; 4 Kennedy Institute of Rheumatology, University of Oxford, Headington, Oxford UK; 5 Centro Tecnológico de Formación Xerencia de Xestión Integrada A Coruña (XXIAC), A Coruña, Spain; 6 CIBERFES, C/Melchor Fernández-Almagro 3, 28029 Madrid, Spain; 7 Universidade da Coruña (UDC), Grupo de Investigación de Reumatología y Salud (GIR-S), Departamento de Fisioterapia, Medicina y Ciencias Biomédicas, Facultad de Fisioterapia, Campus de Oza, A Coruña, Spain

**Keywords:** Rheumatology, Rheumatic diseases, Osteoarthritis

## Abstract

It has been suggested that mitochondrial dysfunction and mtDNA variations may contribute to osteoarthritis (OA) pathogenesis. However, the causative link to support this claim is lacking. Here, we surgically-induced OA in conplastic mice in order to evaluate the functional consequences of mtDNA haplotypes in their joint degeneration. BL/6^NZB^ strain was developed with C57BL/6JOlaHsd nuclear genome and NZB/OlaHsdmtDNA while BL/6^C57^, which is the original, was developed with C57BL/6JOlaHsd nuclear genome and C57/OlaHsdmtDNA for comparison. The surgical DMM OA model was induced in both strains. Their knees were processed and examined for histopathological changes. Cartilage expression of markers of autophagy, apoptosis, oxidative stress and senescence were also analyzed by immunohistochemistry. The joints of BL/6^NZB^ mice that were operated presented more cellularity together with a reduced OARSI histopathology score, subchondral bone, menisci score and synovitis compared to those of BL/6^C57^ mice. This was accompanied with higher autophagy and a lower apoptosis in the cartilage of BL/6^NZB^ mice that were operated. Therefore, the study demonstrates the functional impact of non-pathological variants of mtDNA on OA process using a surgically-induced OA model. Conplastic (BL/6^NZB^ ) mice develop less severe OA compared to the BL/6^C57^original strain. These findings demonstrate that mitochondria and mtDNA are critical targets for potential novel therapeutic approaches to treat osteoarthritis.

## Introduction

Osteoarthritis (OA) is the most common form of arthritis and the leading cause of musculoskeletal disability in the elderly. It affects approximately 250 million people worldwide (including at least 40 million in Europe) and causes severe disability. Pain is the hallmark symptom of OA, and while it is not a lethal disease, it seriously reduces the quality of life and the life expectancy of those suffering from it. The total costs of its treatment induced in EU could be as high as 408 to 817 billion €/year^1,2^. Much of the pathophysiology of OA is yet to be understood, but it is a chronic progressive disorder that involves movable joints and is characterized by cellular stress and extracellular matrix degradation initiated by micro- and macro-injuries. Altogether, it activates maladaptive repair responses, including pro-inflammatory pathways of innate immunity^3^.

The role of mitochondria in the pathogenesis of OA has not been studied in detail until relatively recently, because cartilage has traditionally been classified as highly glycolytic tissue that derives its energy from anaerobic glucose metabolism. However, mitochondrial oxidative phosphorylation (OXPHOS) could account for up to 25% of total adenosine triphosphate (ATP) production in articular cartilage^4^. There is now a growing body of evidence that suggests that mitochondria play a central role in the pathogenesis of OA^5,6^. Mitochondrial dysfunction together with mitochondrial DNA (mtDNA) damage contributes to cartilage degradation via several processes such as: (1) increased apoptosis; (2) enhanced inflammatory responses; (3) decreased mitochondrial biogenesis; (4) increased cartilage catabolism, (5) alterations of mitochondrial dynamics leading to deregulated reactive oxygen species (ROS) production and (6) changes in mitophagy and autophagy mechanisms^7–10^. When these changes cannot be managed by the repair and anti-oxidant systems, the homeostatic system fails, and mitochondrial dysfunction is perpetuated. This leads to progressive cartilage destruction and, eventually, to joint malfunction.

Approximately, between 30 and 65% of the risk of OA is genetically determined^11,12^ with evidence accumulated from different genome-wide association studies (GWAS)^13^. Most of these studies have focused on nuclear genetic variants. However, over the past decade, evidence has accumulated that there is an association between mtDNA haplogroups and the prevalence, progression and incidence of the disease^14–19^. This link has been assessed only in ex vivo studies and only with the use of in vitro transmitochondrial cybrid models^20^. Thus, the causal link between mtDNA haplotype variability and OA joint phenotype has not been studied.

Conplastic mice are a powerful tool to investigate the contribution of mtDNA variants to cell physiology and behaviour^21^. They are developed by backcrossing the nuclear genome from one inbred mouse strain into the cytoplasm of another. These mice have a uniform nuclear background, but different mtDNA variants, thus enabling the study of the function of the mtDNA variants in an in vivo setting. Several strains of conplastic mice have been developed^22^. In a recent study, we showed that the extension of healthy again mice was very dependent on the mtDNA variant^23^. Since OA is an age associated disease, here we have studied conplastic animals to determine whether the substitution of the mtDNA variant would also affect OA development and progression. To induce OA, we used the surgical model of destabilization of the medial meniscus (DMM). It is a post-traumatic osteoarthritis mouse model that can mimic meniscal injury, which is a predisposing factor for OA development in humans. The DMM model allows for the analysis of the structural and biological changes during the disease progression and it is currently considered as a gold standard for the study of post-traumatic OA^24^.

Thus, we developed the posttraumatic OA model and compared the evolution of both strains by histological and immunohistochemistry assays. This study demonstrates that the replacement of mtDNA reduces joint degradation.

## Results

### Development of less severity of cartilage loss and menisci damage by conplastic (BL/6^NZB^) mice after DMM surgery

**Figure 1:**
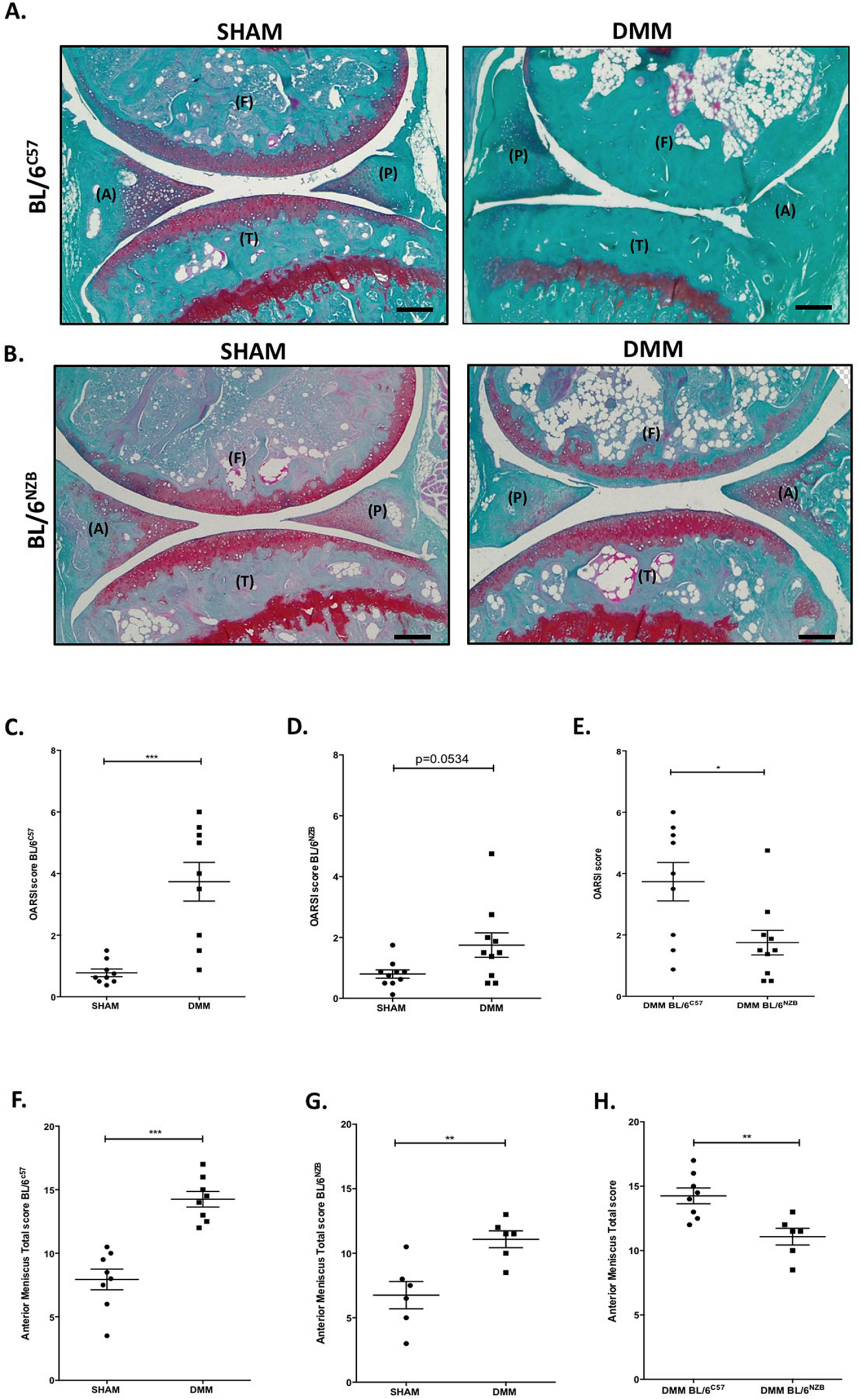
Attenuation of osteoarthritis development in conplastic (BL/6^NZB^) mice following destabilization of the medial meniscus (DMM). Representative Safranin O/Fast green-stained sections of the knee joints (right knee = SHAM; left knee = DMM) of DMM-operated 17-week-old male BL/6^C57^ **(A)** and BL/6^NZB^ **(B)** at 8 weeks after surgery. Images show the medial femorotibial compartment of SHAM or DMM-operated knees. Femur (F), tibia (T) as well as anterior (A) and posterior (P) location of the menisci are also represented (magnification 10x). Scale bar, 500 μm. Quantification of cartilage damage in BL/6^C57^ **(C)** and BL/6^NZB^ **(D)** mice at 8 weeks following DMM is shown as OARSI score (BL/6^C57^ = 9; BL/6^NZB^ = 10), a semi quantitative scoring system where the minimum value 0 corresponds to the normal cartilage and the maximum value 6 represents a cartilage destruction of more that 75% of the articular surface. Comparison of OARSI score between BL/6^C57^ and BL/6^NZB^ mice after DMM surgery **(E)**. Tissue structure (smooth, fibrillation, undulating), cellularity (normal, hyper cellularity, hypo cellularity), and matrix staining of Safranin O/Fast Green (normal and disrupted staining) were scored and summed together for anterior and posterior menisci. Quantification of anterior meniscus total score of BL/6^C57^ **(F)** and BL/6^NZB^ **(G)** after DMM surgery (BL/6^C57^ = 8; BL/6^NZB^ = 6). Comparison of anterior meniscus total score between BL/6^C57^ and BL/6^NZB^ after DMM surgery **(H)**. All data are shown as mean ± SEM; ***p < 0.001, **p < 0.01, *p < 0.05, p = 0.0534 by non-parametric Mann–Whitney test.

Osteoarthritis Research Society International (OARSI) histopathology scoring system was applied to evaluate OA severity. First, the cartilage degradation process in both BL/6^C57^ and BL/6^NZB^ strains was analyzed separately. As shown in Fig. 1, BL/6^C57^ mice presented a severe cartilage loss in joint submitted to the DMM surgery when compared with SHAM joint (Fig. 1A,C, p = 0.0003). However, the induction of OA in BL/6^NZB^ was weaker as reported in panels B and D (Fig. 1B,D, p = 0.0534). Interestingly, comparing the OARSI score of DMM joint of BL/6^C57^ with DMM joint of BL/6^NZB^, it is observed that the development of OA in conplastic mice was significantly less severe than in the original strain (Fig. 1E, p = 0.0180). In addition, there were no differences between the SHAM joints of BL/6^C57^ and BL/6^NZB^ mice (Supplementary Figure S1).

Knee meniscus degeneration and damage are linked to the initiation and progression of osteoarthritis. A semi quantitative grading system was used to study the menisci alterations based on the analysis of tissue structure, cellularity, and matrix staining of Safranin O/Fast Green of both anterior and posterior sections of meniscus. There was a significant increase in the score of anterior meniscus in the operated knees of both BL/6^C57^ (Fig. 1A,F, p = 0.0002) and BL/6^NZB^ (Fig. 1B,G, p = 0.0087) strains compared to SHAM knees. There were no significant differences in posterior menisci (data not shown). SHAM groups had no differences in both anterior and posterior menisci (Supplementary Figure S1).

Moreover, the operated knees of conplastic (BL/6^NZB^) mice showed less menisci degeneration than the original strain; there was statistical significance in the anterior (Fig. 1H, p = 0.0040) but not in the posterior meniscus (data not shown).

### The exacerbation of subchondral bone changes post-DMM surgery in BL/6^C57^ original strain

Together with the progressive cartilage degradation, subchondral bone changes are considered as a hallmark of OA. To study the subchondral bone alterations, a score system was used which evaluated the thickening of the bone, number of the trabeculae and the osteophyte formation of the medial tibial plateau (MTP).

**Figure 2:**
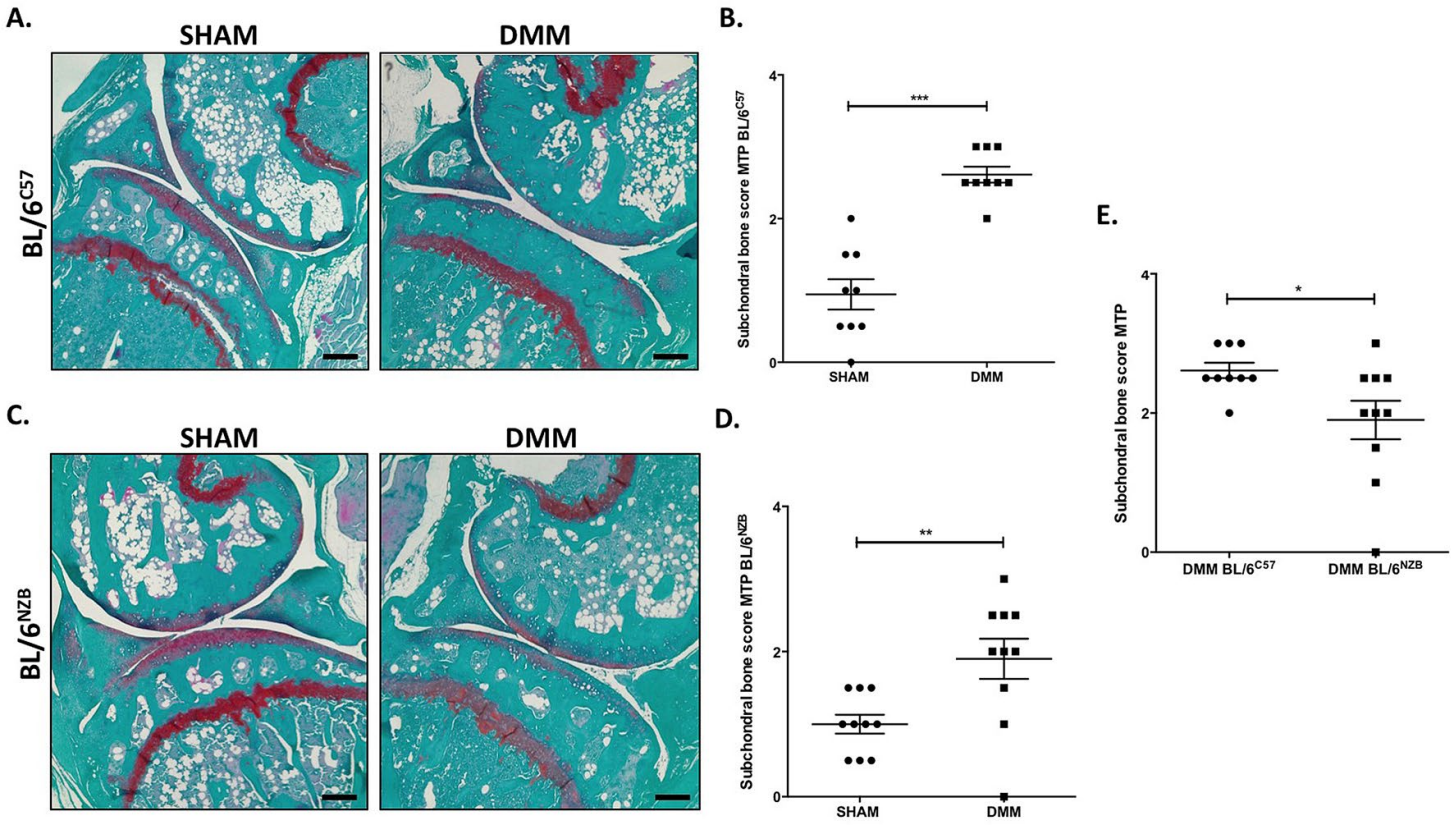
Scoring of subchondral bone changes in tibial plateau from BL/6^C57^ and BL/6^NZB^ operated mice. Representative images of knee joints (right knee = SHAM; left knee = DMM) stained with Safranin O from BL/6^C57^ **(A)** and BL/6^NZB^ **(C)** mice (magnification 10×). Scale bar, 500 μm. Subchondral bone thickening, number of trabeculae and osteophyte formation graded as normal (grade 0), mild (grade 1), moderate (grade 2) or severe (grade 3) were used as parameters to examine the subchondral bone of medial tibial plateau (MTP) in mouse knee joints. Quantification of bone changes in BL/6^C57^ **(B)** and BL/6^NZB^ **(D)** mice at 8 weeks (BL/6^C57^ = 9; BL/6^NZB^ = 10) following DMM is shown as subchondral bone score in medial tibial plateau (MTP). Comparison of bone changes score between BL/6^C57^ and BL/6^NZB^ mice after DMM surgery **(E)**. All data are shown as mean ± SEM; ***p < 0.001, **p < 0.01, *p < 0.05 by non-parametric Mann–Whitney test.

As reported in Fig. 2, in both BL/6^C57^ (Fig. 2A,B, p < 0.0001) and BL/6^NZB^ (Fig. 2C,D, p = 0.0071) strains, there was a significant increase of subchondral bone score in operated knee joint (DMM). In the conplastic (BL/6^NZB^) mice, the operated knee joint presented less subchondral bone changes compared to BL/6^C57^ mice (Fig. 2E, p = 0.0353). There were no differences between SHAM groups (Supplementary Figure S1).

### Reduction of synovitis post DMM surgery in conplastic (BL/6^NZB^) mice

**Figure 3:**
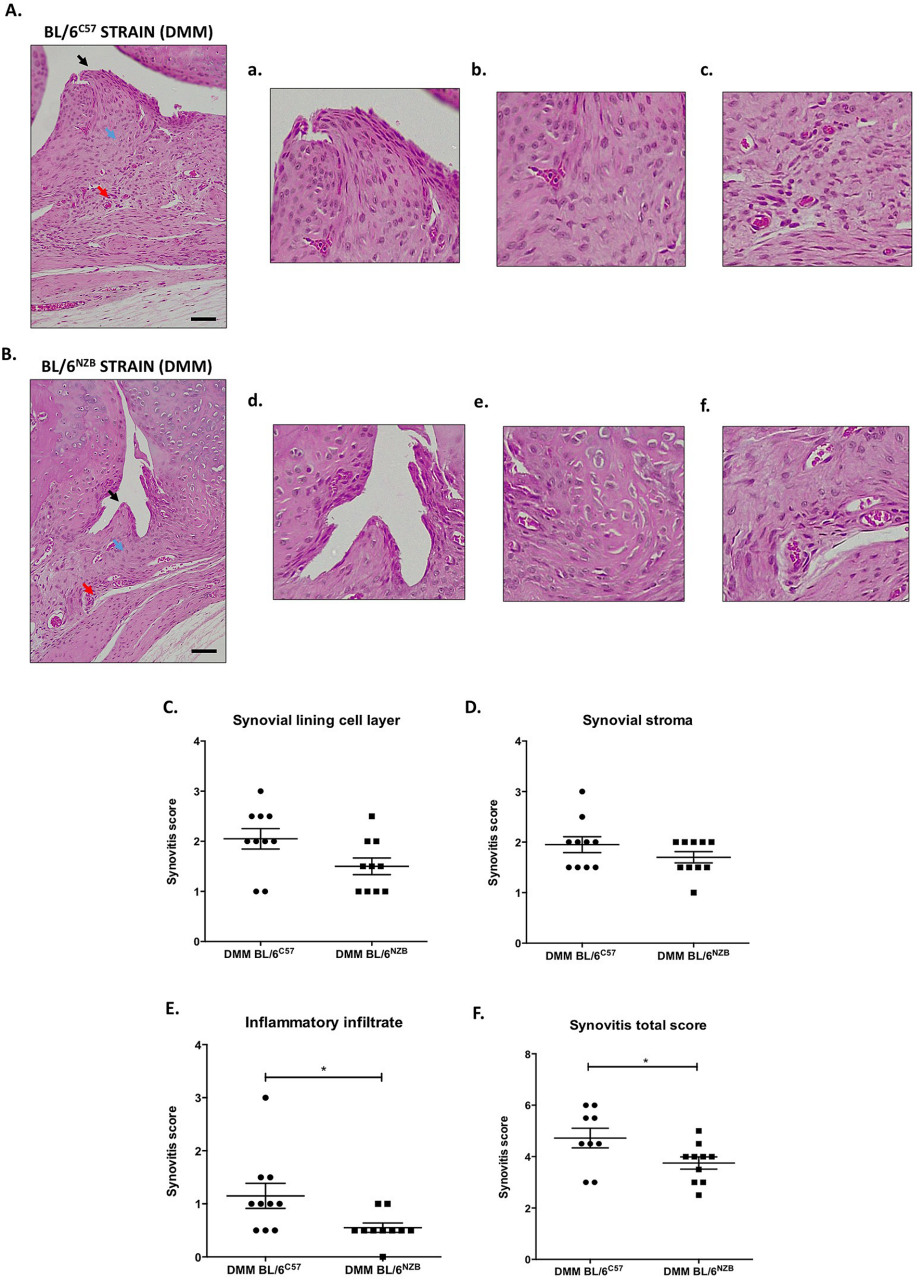
Histological analysis of synovitis in BL/6^C57^ and BL/6^NZB^ operated mice. Representative images of Hematoxylin Eosin (H&E) stained sections from synovium of BL/6^C57^ mice after DMM surgery (**A**, n = 9); synovial lining cell layer (**a**, black arrow), stroma cell density (**b**, blue arrow) and inflammatory infiltrate (**c**, red arrow). Representative images of Hematoxylin Eosin (H&E) stained sections from synovium of BL/6^NZB^ (**B**, n = 10) mice after DMM surgery; synovial lining cell layer (**d**, black arrow), stroma cell density (**e**, blue arrow) and inflammatory infiltrate (**f**, red arrow). Quantification of synovial lining cell layer **(C)**, stroma cell density **(D)** and inflammatory infiltrate **(E)** in BL/6^C57^ and BL/6^NZB^ operated mice. Quantification of synovitis total score in DMM groups from BL/6^C57^ and BL/6^NZB^ mice **(F)**. Original magnification 20×. Scale bar, 250 μm. All data are shown as mean ± SEM; *p < 0.05 by non-parametric Mann–Whitney test. Comparisons with significant differences are indicated; all other comparisons were non-significant.

Synovial abnormalities have been observed at multiple stages of OA. This study measured the synovitis grade by histological analysis of synovial tissues after DMM surgery in joints from BL/6^NZB^ and BL/6^C57^ mice. Three synovial membrane features were evaluated: synovial lining cell layer, stroma cell density and inflammatory infiltrate. In Fig. 3, the synovitis total score was significantly reduced in conplastic (BL/6^NZB^) mice compared to BL/6^C57^ mice after surgery (Fig. 3F). There was a reduction of synovitis score in all the three features analyzed (Fig. 3C,D,E), but there was statistical significance only in the inflammatory infiltration (Fig. 3E). In addition, when the SHAM group was compared with the DMM group, there was a significant induction of synovitis in both strains (Supplementary Figure S2). However, no differences between SHAM groups were observed (Supplementary Figure S2).

### Reduced cartilage cellularity and increased apoptosis displayed in BL/6^C57^ mice submitted to DMM model compared to conplastic (BL/6^NZB^) mice

**Figure 4:**
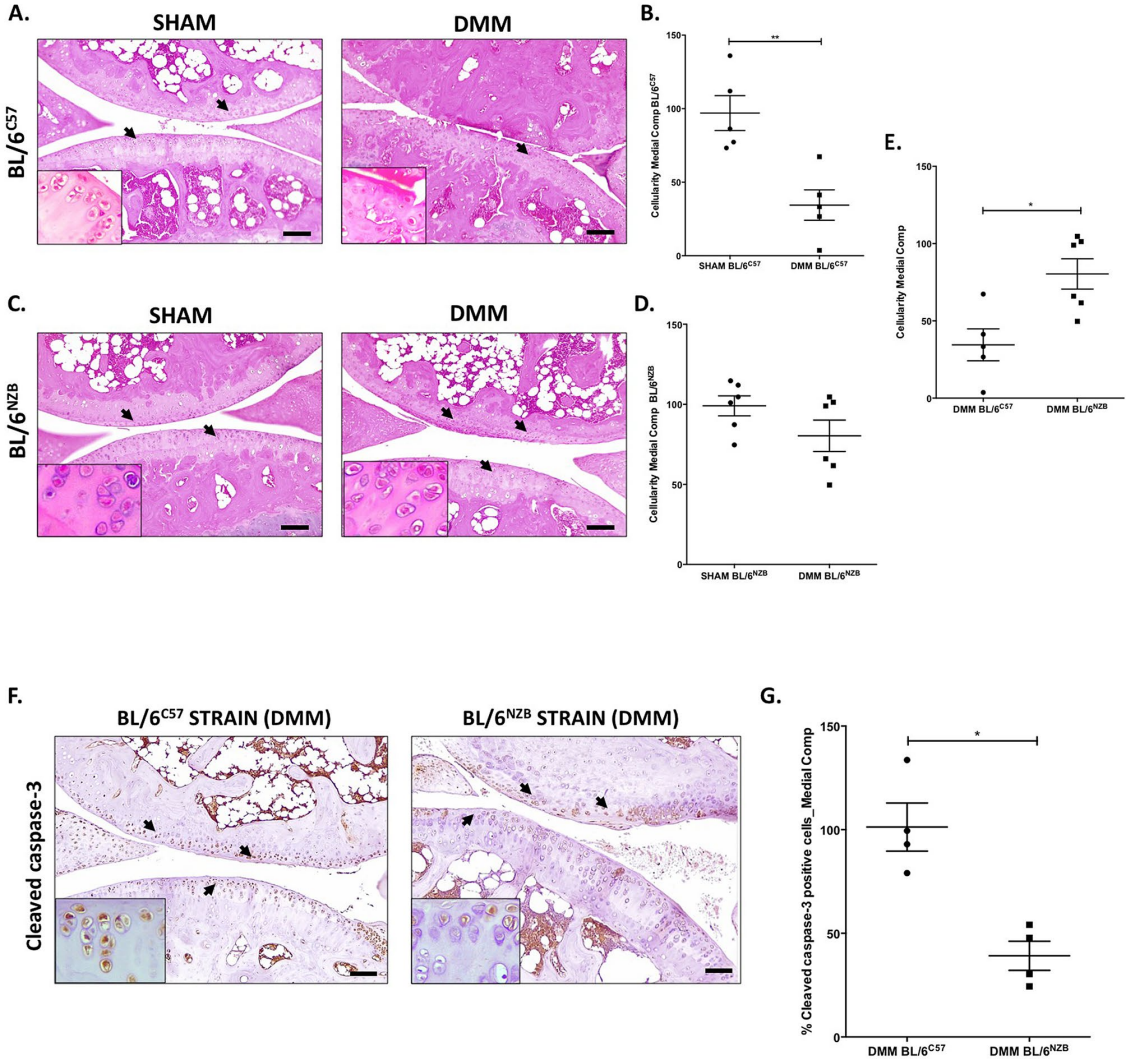
Changes in cartilage cellularity and cleaved caspase-3 expression in response to surgically OA induction. Images from Hematoxylin Eosin (H&E) stained sections of BL/6^C57^ **(A)** and BL/6^NZB^ **(C)** mice knee joints (BL/6^C57^ = 5; BL/6^NZB^ = 6). Original magnification 10×. Scale bar, 500 μm. Chondrocytes magnification (20×) is shown on the bottom-left corner of the images. Quantitative analysis of cartilage cell number in the medial compartment of BL/6^C57^ **(B)** and BL/6^NZB^ **(D)** mice after DMM surgery. Comparison of cartilage cell number between DMM BL/6^C57^ and DMM BL/6^NZB^ mice **(E)**. Representative images of medial compartment of operated (DMM) knee joints from BL/6^C57^ (left panel **F**) and BL/6^NZB^ (right panel **F**) stained with cleaved caspase-3 (CC3) antibody. Quantitative analysis of CC3 positive cells of knee joints from BL/6^C57^(n = 4) and BL/6^NZB^(n = 4) after DMM surgery **(G)**. Original magnification 10×. Scale bar, 500 μm. Black arrow indicate positively stained chondrocyte. Graph represents means ± SEM. *p < 0.05 , **p < 0.01 by non-parametric Mann–Whitney test. Comparisons with significant differences are indicated; all other comparisons were non-significant.

During the OA process, structural changes of cartilage, subchondral bone and menisci are usually accompanied by a reduction of cell density. To evaluate the cartilage cellularity, we quantify the number of chondrocytes in H&E sections from BL/6^C57^ and BL/6^NZB^ after DMM surgery. Figure 4 shows that there was a decrease in DMM knees from BL/6^C57^ mice (Fig. 4A,B, p = 0.0079) and BL/6^NZB^ mice (Fig. 4C,D, p = 0.1429) compared with SHAM knees. However, this difference was statistically significant only in BL/6^C57^ mice. No differences between SHAM knees were observed (Supplementary Figure S3). The cartilage cellularity loss during the development of OA was less severe in conplastic (BL/6^NZB^) mice than in the original strain (Fig. 4E, p = 0.0303).

To determine whether the observed reduction in cartilage cellularity was related to cell death by apoptosis, the cartilage expression of cleaved caspase-3 was measured. After DMM surgery, cartilage from BL/6^NZB^ mice presented significant lower proportion of cleaved caspase-3 (CC3) expression than that of the original strain (Fig. 4F,G, p = 0.0290). There was no detectable difference between the SHAM knees of the two strains (Supplementary Figure S4).

### Autophagy response following cartilage injury

To study the potential molecular modulations in the joints of the mice after being surgically induced with OA, one of the mechanisms that was altered in OA was analyzed: the autophagy.

**Figure 5:**
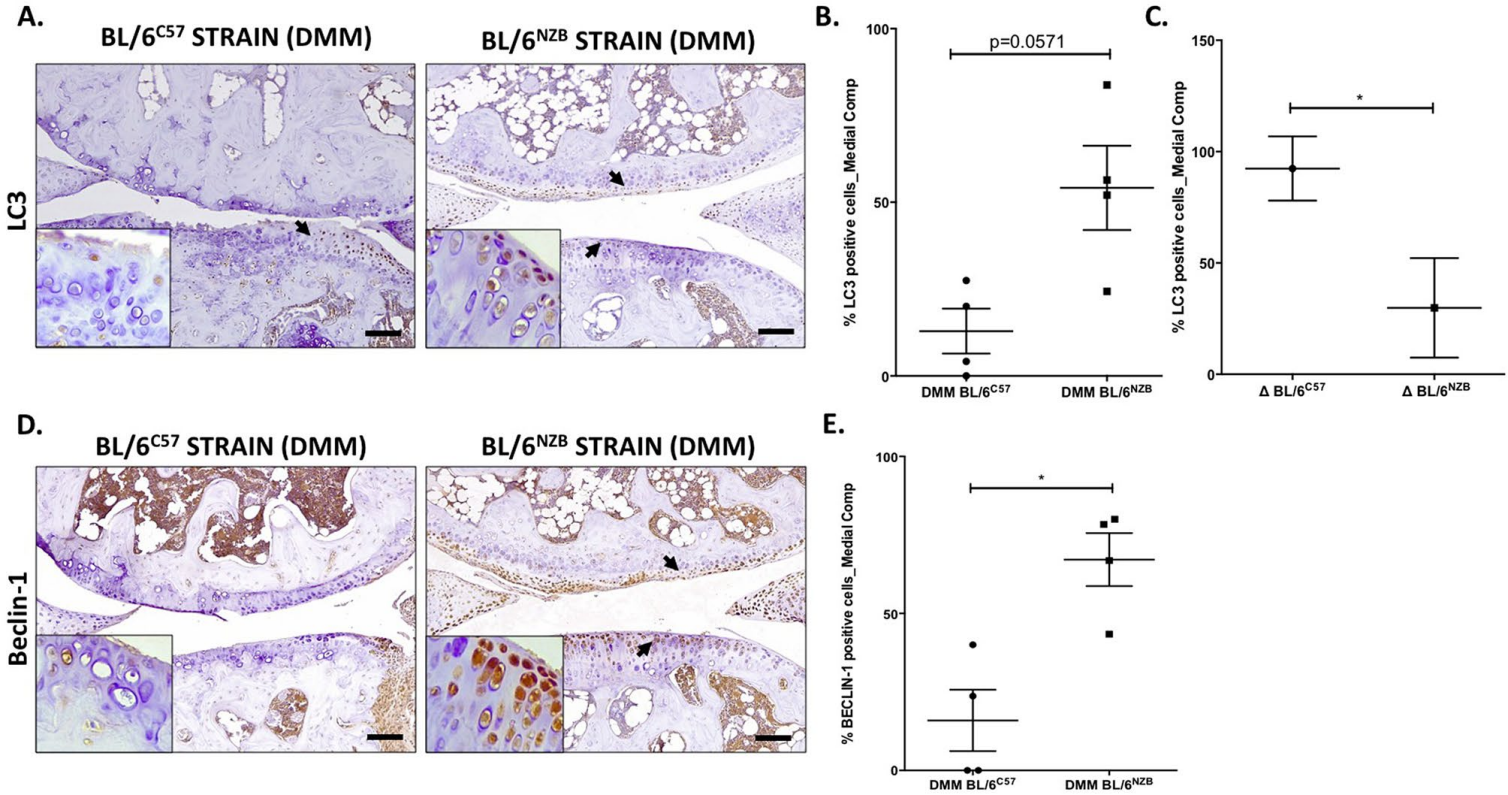
Immunohistochemistry for LC3 and Beclin-1 were performed on mouse knee joint sections. Representative images of medial compartment of operated (DMM) knee joint from BL/6^C57^ (left panel **A**) and BL/6^NZB^ (right panel **A**) stained with LC3. Quantitative analysis of LC3 positive cells of knee joints from BL/6^C57^ and BL/6^NZB^ after DMM surgery **(B)**. Comparison of the difference in LC3 positive cells between SHAM and DMM group (Δ) in joints from BL/6^C57^ and BL/6^NZB^ mice **(C)**. Representative images of medial compartment of operated (DMM) knee joints from BL/6^C57^ (left panel **D**) and BL/6^NZB^ (right panel **D**) stained with Beclin-1 antibody. Quantitative analysis of Beclin-1 positive cells of knee joints from BL/6^C57^ and BL/6^NZB^ after DMM surgery **(E)**. Original magnification 10×. Scale bar, 500 μm. Black arrow indicate positively stained chondrocyte. Chondrocytes magnification (20×) is shown on the bottom-left corner of the images. Graphs represent means ± SEM; (BL/6^C57^ = 4; and BL/6^NZB^ = 4). *p < 0.05, p = 0.0571 by non-parametric Mann–Whitney test.

To address the evaluation of autophagy mechanism in both BL/6^C57^ and BL/6^NZB^ strains, immunohistochemistry of LC3 and Beclin-1 proteins was done. From the histological data, there was more expression of protein LC3 in sections from operated knee BL/6^NZB^ mice when compared with the original strain, that borderlines the statistical significance (Fig. 5A,B, p = 0.0571). Moreover, in comparing the difference in LC3 positive cells between SHAM and DMM groups for each strain, it was found that the loss of LC3 from BL/6^NZB^ mice was significantly less than the one found in cartilage from BL/6^C57^ mice (Fig. 5C, p = 0.0464). The expression of protein Beclin-1 was significantly higher in sections from operated knee BL/6^NZB^ mice than in the original strain (Fig. 5D,E, p = 0.0286). SHAM groups presented no differences for both LC3 and Beclin-1 cartilage expression (Supplementary Figure S5 and S6).

## Discussion

Clinical and in vitro data suggest that specific mtDNA polymorphisms, called mtDNA haplogroups might confer different level of protection/sensitivity to knee OA^11,14–19^. Moreover, several studies have confirmed the link between OA and mtDNA haplogroups. They have reported the association of mtDNA haplogroups with different comorbidities linked to OA^11^, such as obesity^25^, T2DM (type 2 diabetes mellitus)^26^ and oxidative stress^27^.

To explore in vivo the functional effects of non-pathological mtDNA variants during the development of OA, conplastic mice strains (DMM model) were surgically-induced with OA (Supplementary Figure S7). This study shows for the first time that the exchange of mtDNA of C57BL/6JOlaHsd mice with mtDNA from NZB/OlaHsd mice improves the development of OA features in C57BL/6JOlaHsd strain.

The use of conplastic model has emerged as a potent tool to reveal the nature and extent of mtDNA variability. Conplastic mice have a uniform nuclear background, but different mtDNA variants, thus enabling the study of the function of the mtDNA variants in an in vivo setting. This study used two wild type mtDNA variants derived from C57BL/6JOlaHsd (also referred as to BL/6^C57^) and NZB/OlaHsd (BL/6^NZB^). MtDNA from most of the mouse-inbred strain is virtually identical with 1 to 5 single nucleotides of difference, far from the natural variation between two human mtDNA (34 SNPs on average and more than 100 between geographically distant populations)^28^. The mtDNA of the NZB/B1NJ strain is enough different from BL/6J mtDNA to resemble the sequence difference between human mtDNAs of distant populations (about 90 SNPs)^29^. The mtDNAs of C57BL/6 and NZB/OlaHsd mice differ by 12 missense mutations, 4 transfer RNA (tRNA) mutations, 8 ribosomal RNA(rRNA) mutations, and 10 non-coding-region mutations. These correspond with a level of divergence comparable to that between human Eurasian and African mtDNAs^23^.

The proof-of-concept study demonstrates that mtDNA haplotype profoundly influences mitochondrial proteostasis and reactive oxygen species generation, insulin signaling, and ageing parameters including telomere shortening and mitochondrial dysfunction, resulting in profound differences in health longevity between conplastic strains^23^. The study employed these conplastic animals to determine whether the substitution of the mtDNA would also affect the changes in OA development and progression. The study results demonstrate that the mtDNA haplotype determines joint degradation response. Conplastic (BL/6^NZB^) mice developed less severe OA compared with the original strain, BL/6^C57^.

Based on the findings of the pathological changes in all of the joint tissues, OA is considered as a disease of the joint, resulting in “joint failure”. In this work, the key features of OA in the various joint tissues affected were analyzed. The first to be analyzed was the cartilage damage, a key feature in OA pathophysiology; the histological scoring method issued by OARSI^30^ was used to analyze it in conplastic (BL/6^NZB^) mice and was compared with the original strain, BL/6^C57^. The OARSI scores revealed that conplastic (BL/6^NZB^) mice showed less cartilage loss than BL/6^C57^ strain (Fig. 1E). There was a significant increase of OARSI score in DMM group compared to SHAM group only in BL/6^C57^ mice (Fig. 1C).

Menisci are often implicated in OA pathogenesis. Knee meniscus injuries and damage are closely linked to the initiation and progression of OA^31–33^. We also measured the menisci damage after DMM surgery in both strains. A semi quantitative score was calculated to analyze the tissue structure, cellularity, and matrix staining of Safranin O/Fast Green of both anterior and posterior sections. Significant results are obtained only in anterior menisci where the conplastic (BL/6^NZB^) mice developed less menisci alterations compared with the original strain after inducing OA experimentally (Fig. 1H). Anterior menisci score was significantly increased in DMM groups compared with SHAM in both BL/6^NZB^ and BL/6^C57^ mice (Fig. 1F,G). In general, in mice, more changes in cellular and matrix component characterized the anterior menisci than posterior menisci. In addition, the pronounced alterations in the anterior menisci of the DMM model were also likely due to the location of the surgically injury in the medial meniscus^34^.

Further analysis of the features of OA was done, measuring the subchondral bone changes which together with cartilage degradation are widely considered as a hallmark of OA^35^. Subchondral bone thickening, number of trabeculae and osteophyte formation^30^ were used as parameters to examine the subchondral bone of medial tibial plateau (MTP) in both strains. The study findings reported a significant increase of subchondral bone score of MTP in DMM group compared to SHAM group in both strains (Fig. 2B,D). Conplastic (BL/6^NZB^) mice showed less subchondral bone changes in MTP compared to BL/6^C57^ mice after DMM surgery (Fig. 2E).

It is known that abnormal accumulation of mtDNA mutations (mutator mice) induces premature aging and affects musculoskeletal tissues^36,37^. Very recently ,the group of Geurts found that the accumulation of mtDNA mutations in the DNA mutator mouse model predispose to elevated subchondral bone turnover and hypertrophy in calcified cartilage but not to accelerated development of osteoarthritis^38^.

Synovium together with cartilage, bone and menisci plays a critical role in the pathophysiology of OA^39,40^. This study evaluated the abnormalities of synovium by histological analysis using the Kreen grading system^41^. Synovium from conplastic (BL/6^NZB^) mice presented less synovitis compared with BL/6^C57^ mice after DMM surgery (Fig. 3F). Specifically, there was a significant decrease in the inflammatory infiltration of synovium (Fig. 3E). DMM groups from both strains presented more synovitis compared to SHAM groups (Supplementary Figure S2).

Chondrocytes loss is another feature that characterizes OA pathophysiology. So, for completeness, the number of total chondrocytes in both strains was also quantified. In knees with experimental OA, there was a reduction of cartilage cellularity which reached statistical significance only in BL/6^C57^ mice (Fig. 4B,D). In comparing DMM groups from both strains, it was observed that joint cartilages from BL/6^NZB^ presented more chondrocytes compared to the other strain (Fig. 4E).

It is noteworthy that in this animal model consisting of 17 weeks old male mice, there were no differences between SHAM groups in all histological studies (Supplementary Figure S1, S2 and S3), confirming that the dissimilarities between both strains emerged exclusively for the experimental OA induction. These results agree with our study on the role of mtDNA variants in a mouse model of spontaneous OA (manuscript in preparation) where there were no alterations of OA between conplastic (BL/6^NZB^) mice and the original strain during the aging process. It was only when there was a joint damage, we observed a different response between the strains. This could probably be because the repair mechanism or the catabolic responses are different and they lead to a distinct OA response.

Taken together, these histological findings revealed that conplastic (BL/6^NZB^) mice developed a less severe experimental OA, demonstrating that the mtDNA haplotype determines the cartilage, subchondral bone, menisci and synovium severity of alterations in OA joint. These observations motivated further study to examine the probable mechanisms that give rise to these histological changes in the joint. To assess that, we performed immunohistochemistry assay for autophagy, apoptosis, oxidative stress and senescence markers. Two markers of oxidative stress (4-HNE and 8-oxo-dG) and two of senescence (β-Galactosidase and MMP-13) were measured. However, there were no significant differences between both strains for those factors (data not shown).

**Figure 6:**
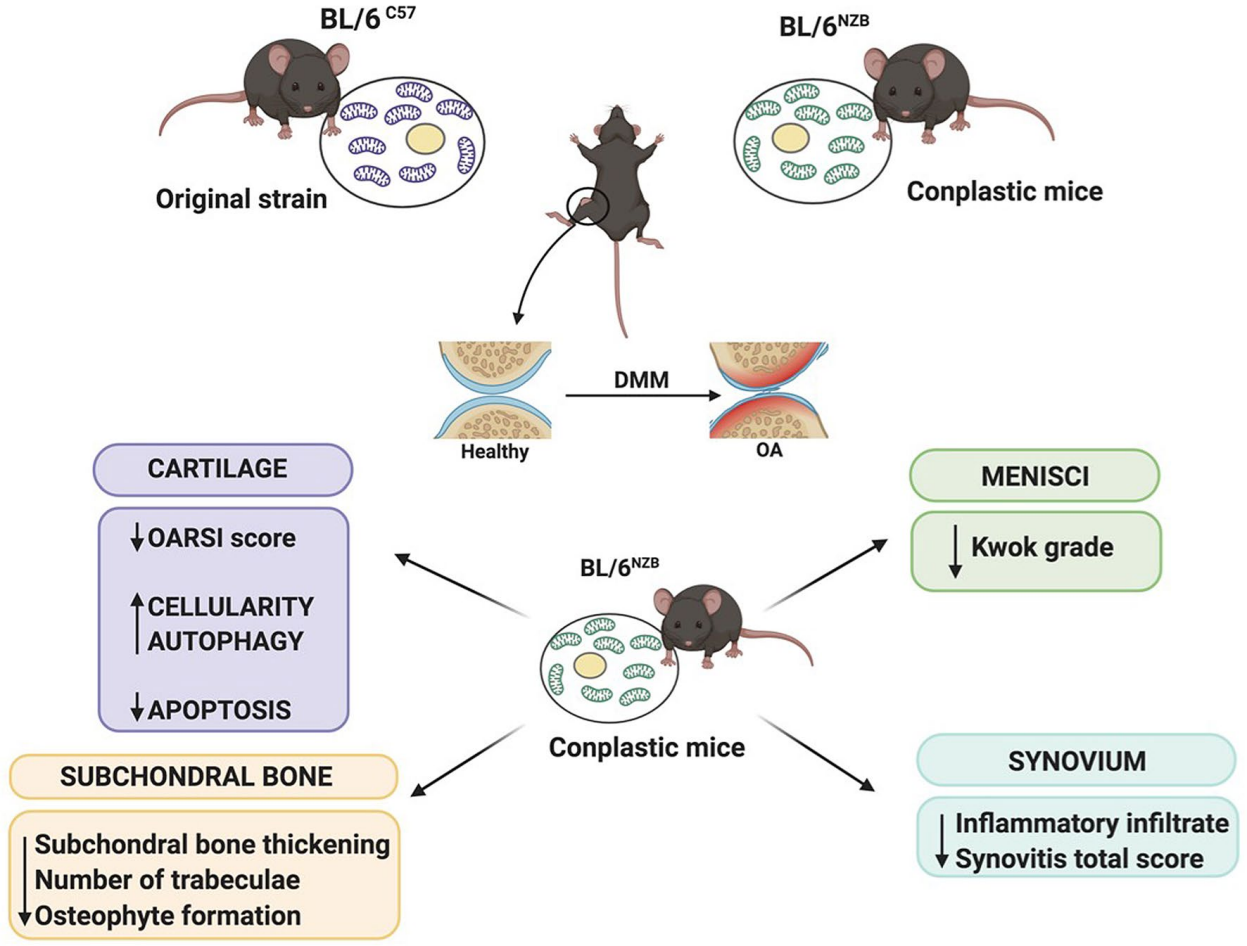
Schematic representation of surgically OA development in conplastic (BL/6^NZB^) mice and BL/6^C57^ strain. Created with BioRender.com.

It has been demonstrated that compromised autophagy may contribute to the development of OA^42,43^, with a reduction of both LC3 and Beclin-1 protein expression in OA chondrocytes^42^. Accordingly, in this study, it was observed a reduced LC3 and Beclin-1 expression in cartilage from DMM groups of both strains reaching statistical significance only in BL/6^C57^ mice for LC3 (Supplementary Figure S5) and in both strains for Beclin-1(Supplementary Figure S6). The decrease of LC3 and Beclin-1 after DMM surgery was significantly less severe in conplastic (BL/6^NZB^) mice than in BL/6^C57^ mice (Fig. 5C,E). Furthermore, given that the reduction of these key regulators of autophagy is accompanied by an increase in apoptosis^42^, we sought to know whether one of the key factors of apoptosis, cleaved-caspase-3, was modulated in cartilage from both strains. Effectively, after DMM surgery, conplastic (BL/6^NZB^) mice showed less CC3 cartilage expression than the original strain (Fig. 4G). The difference in the autophagy and apoptosis responses from conplastic (BL/6^NZB^ ) mice could be related to a greater cartilage cellularity found in those mice after DMM surgery when compared with BL/6^C57^ strain (Fig. 4E). Thus, the reduction of OA severity in conplastic (BL/6^NZB^ ) mice seems to be mediated by a different induction of autophagy and specially a lower apoptosis which probably is responsible for the different response to the surgically OA induction (Fig. 6).

A limitation of this study is that the molecular pathways which could be implicated in the differential response of knee joint to OA induction in the mice strains analyzed were not investigated. Therefore, this is a descriptive study without previous data on the mice joint before OA induction. This aspect would give a more detailed information on the development of the OA pathophysiology in the different mice strains.

The study results demonstrated that substituting the two wild type mtDNA variants affects the joint deterioration, ameliorating the development of OA. In addition, more autophagic response and lower apoptosis induction could explain the high cellularity observed in the cartilage and mediated the less severe OA induction in conplastic (BL/6^NZB^) mice (Fig. 6). The demonstration of the causal relationship between mtDNA variants and OA highlights that mitochondria should be prominently focused on to develop therapeutic targets and to properly stratify patients’ risk to identify the right level of care for distinct patients.

## Materials and methods

### Animals

Conplastic mice strain were generated in the laboratory of Dr. José Antonio Enríquez^23^ by backcrossing females (mitochondrial donors) with males of the parental recipient strain. Parental C57BL/6JOlaHsd and NZB/OlaHsd strains were purchased from Harlan Laboratories.

Specifically, conplastic (BL/6^NZB^) mice strain was developed with the C57BL/6JOlaHsd nuclear genome and NZB/OlaHsdmtDNA to compare with the original C57BL/6JOlaHsd strain (BL/6^C57^).

Mice were bred at the Experimental Surgery Unit of A Coruña under standard conditions (12:12 light: dark cycle, + 22 ± 1 °C temperature, 50–60% humidity); and food and water provided ad libitum. After the experiments, the animals were sacrificed by carbon monoxide followed by cranial dislocation.

Animal experiments were carried out in accordance with the legislation for the protection of animals (European Directive 2010/ 63) used for scientific purposes, and in compliance with the ARRIVE guidelines. The study was approved by the Local Ethical Committee of the Animal Experimentation “Comité de Ètica de Experimentación Animal de la Xerencia de Xestión Integrada A Coruña (CEEA-XXIAC)” and by the “Consellería do Medio Rural” of Xunta de Galicia (15002/2018/01).

### DMM-induced OA model

The surgical OA model was induced in 17 weeks old BL/6^C57^ (n = 10) and BL/6^NZB^ (n = 10) male mice by the transection of the medial meniscotibial ligament (DMM: The surgical model of destabilization of the medial meniscus) of the hind left knee joint.

In brief, the joint capsule was exposed by cutting the skin 4 mm. The medial meniscus was displayed after opening the joint capsule and the meniscotibial ligament was transected. To close the joint capsule and skin we used a 7-0 sutures. The contralateral knee that was not operated was used as a control (sham). Analgesic (Buprenorphine 0.09 mg/kg sc) and antibiotic (Oxacillin, orally) were administered during the surgery and for 3 days (analgesic) and 7 days (antibiotic) post-surgery. The animals were euthanized 8 weeks later (Supplementary Figure S7).

### Histological analysis

Knee joints from mice of DMM-induced OA model were harvested. Joints were fixed overnight in 3.7–4% formaldehyde and then decalcified in Decal (HistoLab) for 6 h at 100 rpm. This was followed by paraffin embedding. Serial sections (4 μm) were cut, deparaffinized in xylene, and washed in a graded series of alcohol.

To analyze the cartilage deterioration in the joint from the DMM model, sagittal sections from right (sham) and left (operated) hind knee joints of BL/6^NZB^ and BL/6^C57^ mice were stained with Safranin O-fast green. They were examined for histopathological changes using the histological scoring method issued by OARSI. This is a semi quantitative scoring system where the minimum value 0 corresponds to the normal cartilage and the maximum value 6 represents a cartilage destruction of more than 75% of the articular surface^30^.

Subchondral bone thickening, number of trabeculae and osteophyte formation graded as normal (grade 0), mild (grade 1), moderate (grade 2) or severe (grade 3) were used as parameters to examine the subchondral bone of medial tibial plateau (MTP) in the knee joints of the mice^30^.

Meniscal damage was scored following the histological grading system published by Kwok et al.^34^. The total scores for all criteria tissue structure (smooth, fibrillation, undulating), cellularity (normal, hyper cellularity, hypocellularity), and matrix staining of Safranin O/Fast Green (normal and disrupted staining) were summed together for anterior and posterior menisci.

Grading of synovial membranes was carried out on routine hematoxylin and eosin (H&E)-stained slides. Three synovial membrane features were evaluated: synovial lining cell layer, stroma cell density and inflammatory infiltrate; they were graded as normal (0), slight (1) moderate (2) and severe (3)^41^.

Sections were blinded and scored by two different experienced scientists. Average scores were used in the statistical analyses.

### Cartilage cellularity

To analyze the cartilage cellularity, knee joint sections were stained with Hematoxylin and Eosin. Three pictures of the center of femoral condyle and tibial plateau of the medial compartment that is not covered by the menisci were taken under 40× magnification. Then, the total number of chondrocytes was counted in each section^44^.

### Immunohistochemistry

Mouse knee joint sections were deparaffinized, washed, and incubated with primary antibody to LC3 (PM036; MBL; 1:1000), Beclin-1 (ab 62557; Abcam; 1:200) or cleaved caspase-3 (CC3) (#9664; Cell Signaling; 1:100), 1 h at room temperature. This was followed by the Dako REAL EnVision Detection System, Peroxidase/DAB + (K-5007, Dako). Finally, the slides were mounted with DePEX mounting medium (Leica Biosystem).

In both right and left mouse knee joints, three pictures of the center of medial femoral condyle and medial tibial plateau that is not covered by the menisci were taken using a microscope (LEICA ICC50W). The total number of chondrocytes and the number of positive cells for the corresponding antibody were counted in each section. The values are expressed as % of positive cells vs total number of chondrocytes^44^.

### Statistical analysis

The results are given as mean ± standard error of the mean (SEM); statistical analysis was performed using the non-parametric Mann–Whitney test. P values less than 0.05 were considered significant. Data were analyzed using the Prism computerized package (Graph Pad Prism Software v 6.0, San Diego, CA, USA).

## Supplementary Information

Supplementary Figures.
