# Supplementary material for: Mitochondrial DNA impact on joint damaged process in a conplastic mouse model after being surgically induced with osteoarthritis
Source: Sci Rep. 2021 Apr 27;11:9112. doi: 10.1038/s41598-021-88083-0 (PMC8079696; doi:10.1038/s41598-021-88083-0)
Supplement: Supplementary file 1 — Supplementary Figures. [file 41598_2021_88083_MOESM1_ESM.docx]

**Mitochondrial DNA impact on joint damaged process in a conplastic mouse model after being surgically induced with osteoarthritis**

**Morena Scotece^1^,** Ignacio Rego-Pérez^1^, Ana Victoria Lechuga-Vieco^2,3,4^,Alberto Centeno Cortés^5^, María Concepción Jiménez Gómez^2^, Purificación Filgueira-Fernández^1^, Carlos Vaamonde-García^1^, José Antonio Enriquez^2,6^, Francisco J. Blanco^1,7*^

1) Grupo Investigación en Reumatología (GIR), Servicio de Reumatología. Instituto de Investigación Biomédica de A Coruña (INIBIC). Complexo Hospitalario Universitario de A Coruña (CHUAC), A Coruña, España.

2) Centro Nacional de Investigaciones Cardiovasculares Carlos III, Madrid, Spain.

3) CIBERES: C/ Melchor Fernández-Almagro 3, 28029 Madrid, Spain.

4) Kennedy Institute of Rheumatology, University of Oxford, Headington, Oxford, UK

5) Centro Tecnológico de Formación Xerencia de Xestión Integrada A Coruña (XXIAC), A Coruña, España

6) CIBERFES: C/ Melchor Fernández-Almagro 3, 28029 Madrid, Spain

7) Grupo de Investigación en Reumatologia-Salud, Departamento de Fisioterapia, Medicina y Ciencias Biomédicas, Agrupación CICA-INIBIC, Universidad de A Coruña, A Coruña, Spain.

*Corresponding author: [fblagar@sergas.es](mailto:fblagar@sergas.es)

**Supplementary Figures**

**Supplementary Figure S1. Measurement of OARSI score, subchondral bone and menisci changes in SHAM groups.**

Representative Safranin O/Fast green-stained sections of the right knee joints (SHAM) of DMM-operated 17-week-old male BL/6^C57^ (n=9) and BL/6^NZB^ (n=10) at 8 weeks after surgery. Images show the medial femorotibial compartment of SHAM knee using 10x magnification (A and C). Scale bar, 500 μm. Quantification of cartilage damage in BL/6^C57^ and BL/6^NZB^ mice is shown as OARSI score, a semi quantitative scoring system where the minimum value 0 corresponds to the normal cartilage and the maximum value 6 represents a cartilage destruction of more that 75% of the articular surface. Comparison of OARSI score between SHAM knees from BL/6^C57^ and BL/6^NZB^ mice (B).

Quantification of bone changes in BL/6^C57^ and BL/6^NZB^ mice is shown as subchondral bone score in medial tibial plateau (MTP). Subchondral bone thickening, number of trabeculae and osteophyte formation graded as normal (grade 0), mild (grade 1), moderate (grade 2) or severe (grade 3) were used as parameters to examine the subchondral bone of medial tibial plateau (MTP) in mouse knee joints. Comparison of bone changes score between SHAM knees from BL/6^C57^ and BL/6^NZB^ mice (D).

Representative images of BL/6^C57^ and BL/6^NZB^ right knee joints showing the femur (F), tibia (T) as well as anterior (A) and posterior (P) location of the menisci (Safranin O staining) in SHAM knees (BL/6^C57^ = 8; BL/6^NZB^ = 6) (panel A). Tissue structure (smooth, fibrillation, undulating), cellularity (normal, hyper cellularity, hypo cellularity), and matrix staining of Safranin O/Fast Green (normal and disrupted staining) were scored and summed together for anterior and posterior menisci. Quantification and comparison of anterior (B) and posterior (C) meniscus total score between SHAM knees from BL/6^C57^ and BL/6^NZB^ mice. 10x magnification. Scale bar, 500 μm. All data are shown as mean ± SEM.

**Supplementary Figure S2. Synovitis measurement in BL/6^C57^ and BL/6^NZB^ strains.**

Images from Hematoxylin Eosin (H&E) stained sections of synovium form BL/6^C57^ (A) and BL/6^NZB^ (B) mice (SHAM BL/6^C57^ = 10; DMM BL/6^C57^ = 9; SHAM BL/6^NZB^ = 10; DMM BL/6^NZB^ = 10). Magnification 20x. Scale bar, 250 μm. Quantification of synovitis total score in synovium from SHAM and DMM groups of BL/6^C57^ (C) and BL/6^NZB^ (D) mice. Quantification of synovitis total score in SHAM groups from BL/6^C57^ and BL/6^NZB^ mice (E). All data are shown as mean ± SEM; *p<0.05, ***p<0.001 by non-parametric unpaired t-test Mann-Whitney. Comparisons with significant differences are indicated; all other comparisons were non-significant.

**Supplementary Figure S3. Cellularity measurement in SHAM groups.**

Images from Hematoxylin Eosin (H&E) stained sections of BL/6^C57^ and BL/6^NZB^ right knee joints. (BL/6^C57^ = 5; BL/6^NZB^ = 6) (A). Magnification 10x. Scale bar, 500 μm. Quantitative analysis of cartilage cell number in the medial compartment (Medial Comp) of SHAM knees from BL/6^C57^ and BL/6^NZB^ (B) mice after DMM surgery. Comparison of cartilage cell number between SHAM BL/6^C57^ and SHAM BL/6^NZB^ mice (B). All data are shown as mean ± SEM.

**Supplementary Figure S4. Cleaved caspase-3 expression in mouse SHAM knees.**

Representative images of medial compartment of SHAM and DMM knee joints from BL/6^C57^ and BL/6^NZB^ stained with CC3 (A). Quantitative analysis of CC3 positive cells of SHAM knee compared with DMM group from BL/6^C57^ (B) and from BL/6^NZB^ mice (C). Quantitative analysis of CC3 positive cells of SHAM knees joints from BL/6^C57^ and BL/6^NZB^ mice (D). Original magnification 10x. Scale bar, 500 μm. Graphs represent means ± SEM; (BL/6^C57^ = 4; and BL/6^NZB^=4).

**Supplementary Figure S5. LC3 cartilage expression in BL/6^C57^ and BL/6^NZB^ strains.**

Representative images of medial compartment of SHAM and DMM knee joints from BL/6^C57^ and BL/6^NZB^ stained with LC3 (A). Quantitative analysis of LC3 positive cells of SHAM knee compared with DMM group from BL/6^C57^ (B) and from BL/6^NZB^ mice (C). Quantitative analysis of LC3 positive cells of SHAM knees joints from BL/6^C57^ and BL/6^NZB^ mice (D). Original magnification 10x. Scale bar, 500 μm. Graphs represent means ± SEM; (BL/6^C57^ = 4; and BL/6^NZB^=4).

**Supplementary Figure S6. Beclin-1 cartilage expression in BL/6^C57^ and BL/6^NZB^ strains**

Representative images of medial compartment of SHAM and DMM knee joints from BL/6^C57^ and BL/6^NZB^ stained with Beclin-1 (A). Quantitative analysis of Beclin-1 positive cells of SHAM knee compared with DMM group from BL/6^C57^ (B) and from BL/6^NZB^ mice (C). Quantitative analysis of Beclin-1 positive cells of SHAM knees joints from BL/6^C57^ and BL/6^NZB^ mice (D). Original magnification 10x. Scale bar, 500 μm. Graphs represent means ± SEM; (BL/6^C57^ = 4; and BL/6^NZB^=4).

**Supplementary Figure S7. Schematic representation of surgically OA model (DMM)**
